# Asthma Care in Resource-Limited Settings: Closing the Treatment Gap Through Access, Innovation, and Action

**DOI:** 10.7759/cureus.113563

**Published:** 2026-07-29

**Authors:** Pradeep K Verma

**Affiliations:** 1 Community Medicine, Mansarovar Medical College and Mansarovar Global University (MGU) Hospital, Sehore, IND

**Keywords:** asthma, asthma management, chronic respiratory disease, health equity, health policy

## Abstract

Asthma is one of the most common chronic diseases worldwide, yet its burden falls most heavily on people living in low- and middle-income countries (LMICs), where the majority of asthma-related deaths occur. This inequity is driven largely by systemic barriers: the limited availability and frequent unaffordability of inhaled corticosteroids, weak primary care infrastructure, diagnostic gaps, over-reliance on reliever medication and oral steroids, and environmental exposures that worsen disease. The result is a striking paradox, because the tools needed to control asthma are relatively inexpensive and effective, yet they fail to reach those who need them most. This editorial reflects on the principal challenges facing asthma care in resource-limited settings, considers emerging innovations that offer reasons for optimism, and outlines priorities for the future. Promising developments include simplified treatment strategies that combine controller and reliever therapy, pooled procurement to lower medicine prices, task-shifting that extends care to nurses and community health workers, integration of asthma management into existing primary care platforms, and digital health tools that support adherence and remote follow-up. Their feasibility varies across settings and depends on adequate training, supervision, and infrastructure. Closing the treatment gap will require simultaneous action on medicine access, health-system strengthening, locally adapted guidelines, and greater political attention to chronic respiratory disease, which is intensifying as climate change worsens air quality and lengthens allergy seasons. Equitable asthma care is not an aspiration beyond our means but a deliverable goal that demands sustained commitment from clinicians, policymakers, and the wider health community.

## Editorial

Asthma is one of the most common chronic diseases in the world, and it is also one of the most inequitably managed. Global estimates indicate that asthma affects several hundred million people, and the majority of asthma-related deaths occur in low- and middle-income countries (LMICs) [1]. Although the condition is treatable with well-established therapies that are relatively inexpensive, the gap between what is achievable and what is delivered remains wide in resource-limited settings. This is a troubling indictment of health inequity, because a large share of asthma deaths are, in principle, preventable. The central paradox of asthma care today is that we already possess the tools to control the disease; what we lack is a reliable way to deliver them to everyone who needs them. It is important, however, to recognize that LMICs are not a uniform group. Medicine availability, health workforce capacity, diagnostic resources, and financing arrangements differ markedly between and within countries, and any strategy must be adapted to local realities rather than applied as a single template.

The foremost barrier is access to essential control medication. Inhaled corticosteroids are the cornerstone of asthma management, yet in many low-resource settings they are frequently unavailable in public facilities and, where available, are often unaffordable for ordinary households [2]. In the absence of controllers, patients and clinicians often default to short-acting relievers for symptom relief and to repeated courses of oral steroids during flare-ups. This pattern eases symptoms in the short term but leaves the underlying airway inflammation untreated, driving avoidable illness, cumulative steroid harm, and death. Reliever-only care may feel like a solution at the bedside, but it perpetuates a cycle of exacerbation and hospital visits that a controller inhaler could prevent.

Diagnosis presents a parallel challenge. Objective testing of lung function, such as spirometry and peak flow measurement, is central to standardized, guideline-based treatment, yet it is scarce outside larger centers, and many frontline health workers have limited training in its use or interpretation. Where such testing is available, it allows asthma to be confirmed, severity to be graded, and treatment to be stepped up or down with confidence; where it is absent, care necessarily relies more heavily on clinical history and symptom patterns. As a result, asthma is often missed, mislabeled as a recurrent chest infection, or confused with other respiratory conditions, while some patients are treated for asthma they do not have. Weak primary care systems, fragmented supply chains, and a shortage of trained personnel compound these problems. Environmental drivers such as indoor smoke from cooking fuels, outdoor air pollution, occupational exposures, and tobacco intensify disease severity in exactly the populations least able to obtain care. These environmental pressures are being amplified by climate change, which lengthens and intensifies pollen seasons, worsens air quality, and increases the frequency of extreme weather events, adding a further and growing burden for people with asthma in the settings least equipped to respond.

Encouragingly, the landscape is beginning to shift, and there are real reasons for optimism. Simplified treatment strategies that place anti-inflammatory therapy at the center of care, including combined controller and reliever therapy within a single inhaler, are well-suited to settings where follow-up is difficult and adherence to separate maintenance and rescue inhalers is low [3]. Such approaches reduce complexity for both patients and prescribers, though their benefit still depends on the underlying medicines being reliably available and affordable, which is not yet the case everywhere. When treatment is easier to follow and supplies are secure, control improves, and dangerous flare-ups become less frequent.

Supply-side and delivery innovations matter just as much. Pooled and regional purchasing can lower prices and stabilize supply, while including asthma medicines in national essential medicines lists and insurance packages improves affordability at the point of care. Task-shifting, in which nurses, community health workers, and pharmacists are trained to diagnose, initiate, and monitor treatment using structured protocols, can extend care well beyond the reach of specialists [4]. For task-shifting to be safe and effective, it must be supported by adequate training, ongoing clinical supervision, clear referral pathways for severe or uncertain cases, and attention to patient safety, rather than simply transferring responsibility to less specialized staff. Integrating asthma and chronic respiratory disease management into existing primary care and non-communicable disease services offers an efficient route to scale without building costly parallel systems. Digital tools add a further dimension: reminders, decision support, and remote follow-up can strengthen adherence and guide frontline workers. Their reach, however, is constrained by digital literacy, device ownership, electricity, and reliable internet access, all of which are uneven in resource-limited settings, so these tools should complement rather than replace medicines, trained staff, and functioning health systems.

Looking ahead, closing the asthma care gap requires action on several fronts at once. Universal availability and affordability of quality controller inhalers must be treated as a public health priority so that no patient is forced to choose between an inhaler and other basic needs. Health systems need investment in primary care capacity, health-worker training, and reliable supply chains, alongside guidelines adapted to local realities rather than transplanted from well-resourced settings. Stronger, context-specific research is essential to inform sound policy, and asthma must be given the political attention it deserves within the broader agendas of non-communicable disease and universal health coverage. For too long, chronic respiratory disease has been overshadowed despite its substantial and rising burden.

The tools to control asthma are affordable and effective; the moral and public health imperative now is to ensure they reach everyone, everywhere. Equitable asthma care is not an aspiration beyond our means. It is a deliverable goal, and meeting it will depend on the sustained and shared commitment of clinicians, policymakers, and the wider health community.
